# Heart Failure in a Young Adult with a Fine-Lubinsky Syndrome: An Unknown Comorbidity

**DOI:** 10.1155/2024/5596010

**Published:** 2024-01-02

**Authors:** Blake Purtle, Jason Wagner, Andrew Zarker, Vishal Patel, David Aguilar

**Affiliations:** ^1^Department of Internal Medicine, University of Texas Health Science Center at Houston, 6431 Fannin, MSB 1.150, Houston, TX, USA; ^2^Cardiovascular Medicine, Department of Internal Medicine, University of Texas Health Science Center at Houston, 6431 Fannin, MSB 1.150, Houston, TX, USA

## Abstract

The Fine-Lubinsky syndrome (FLS) is a rare congenital disorder. Heart failure has not been described in young adults with this condition. Here, we report the first case of heart failure in a young adult patient with FLS. This finding highlights the need for further investigation into cardiac complications in this illness.

## 1. History of Presentation

A 25-year-old man presented to clinic with his mother with two weeks of shortness of breath, dyspnea on exertion, and lower extremity swelling. Prior to admission, he was able to walk almost 2 miles daily without difficulties; however, recently, he was only able to walk one mile per day, limited by fatigue. Additionally, he also reported a dry, nonproductive cough for approximately one month. His mother denied symptoms of fever, myalgias, chest pain, recent travel, or contact with sick persons. COVID-19 testing done before admission had been negative. A chest X-ray was performed that demonstrated cardiomegaly, and he was referred to the ER for further evaluation.

On physical exam, the patient was afebrile and had nonlabored breathing, blood pressure was 106/74 mmHg, and his heart rate was 106 beats/min. The patient was found to have elevated jugular distension, an S3 on auscultation, a distended abdomen, and trace pitting edema in the lower extremities.

## 2. Past Medical History

Past medical history included the Fine-Lubinsky syndrome, diagnosed at age 11. His manifestations include cranial abnormalities, auditory problems that subsequently resolved with age, intellectual disability, and speech impairment. The patient required multiple intracranial surgeries, including resection of intracranial fibrous dysplasia [1] at the age of 11. His family history was notable for hypertension in his mother, father, and grandparent. His grandfather had a heart attack in his 30s along with heart failure. His mother denied that the patient had ever smoked and used illicit drugs or alcohol. He did not have hypertension or diabetes.

## 3. Differential Diagnosis

Heart failure in young adults carries a unique differential diagnosis from that of older adults, where ischemic heart disease is the most common etiology. In young adults, the most common etiology is idiopathic dilated cardiomyopathy (DCM), followed by ischemic heart disease and hypertension [2]. Given his age, congenital heart defects and substance abuse (such as cocaine, methamphetamines, and anabolic steroid use) also need to be considered [3].

## 4. Investigation

**Figure 1:**
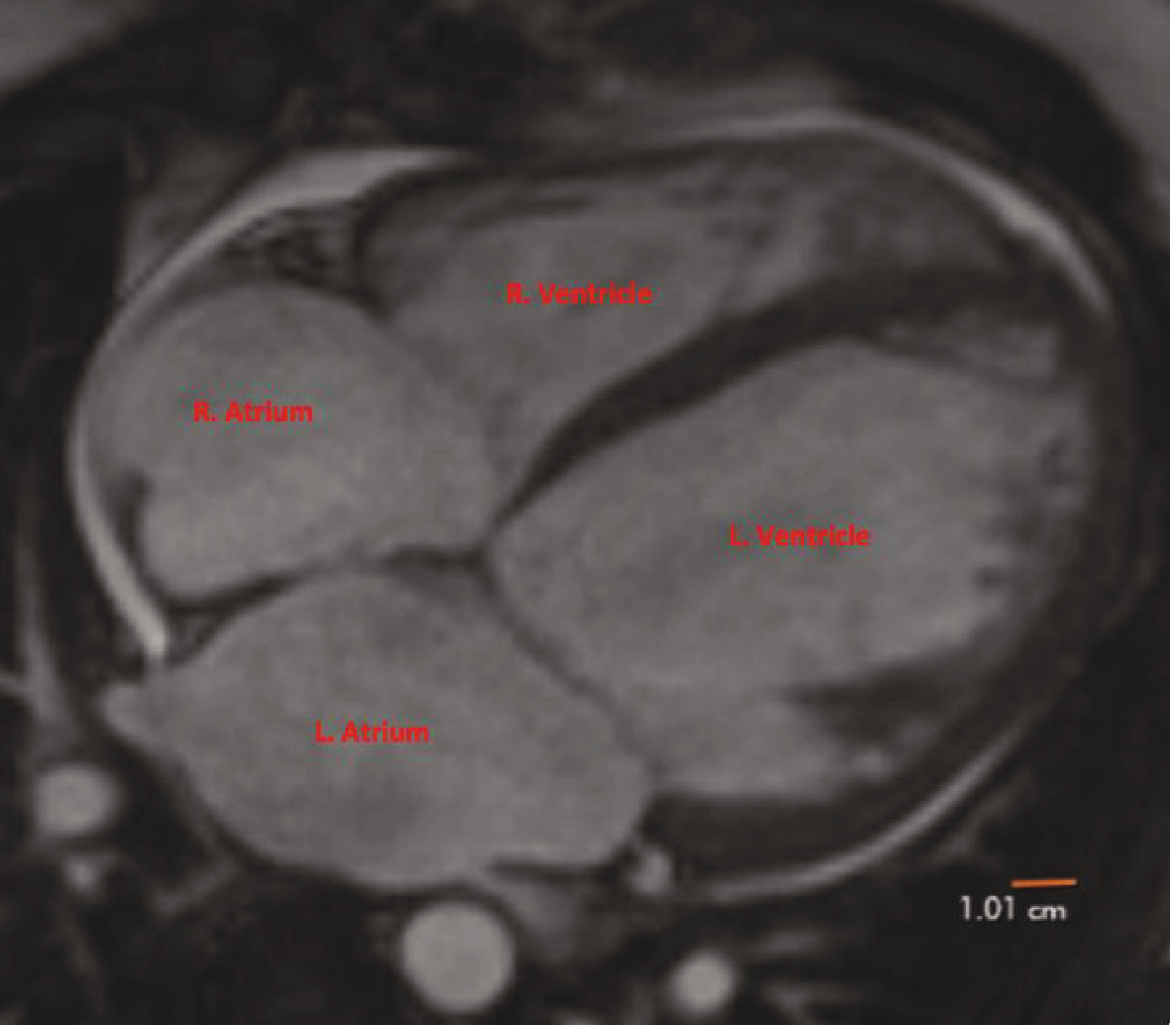
Cardiac MRI showing normal wall thickness and dilated right and left atria and dilated right and left ventricles. The red bar is a standard reference ruler. Markers are placed to indicate the anatomical locations of the ventricles and atria.

**Figure 2:**
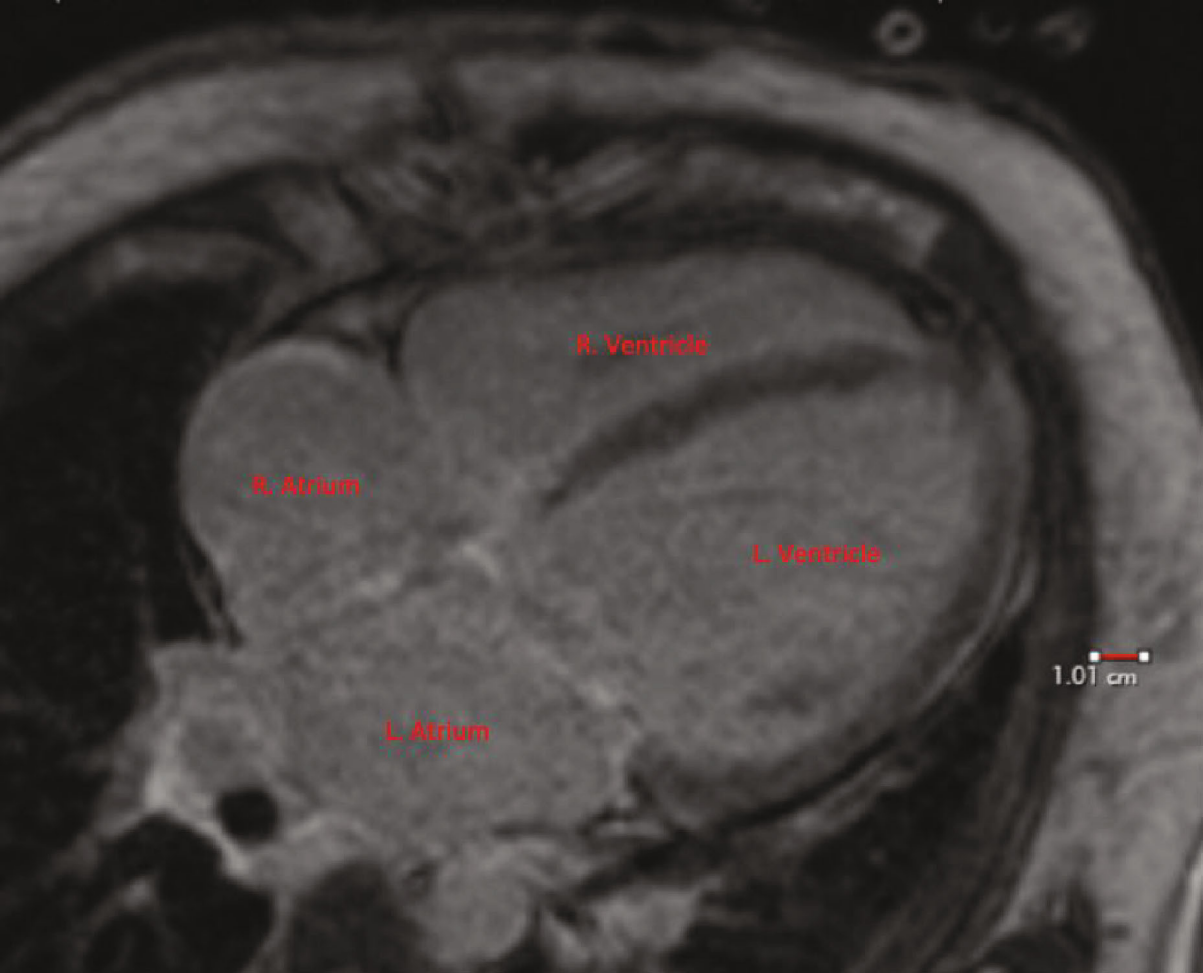
Cardiac MRI showing normal enhancement on delayed gadolinium-enhanced imaging. The red bar is a standard reference ruler. Markers are placed to indicate the anatomical locations of the ventricles and atria.

On admission, his labs were notable for a brain natriuretic peptide of 2,878 pg/mL and troponin <0.02 ng/mL. A thyroid panel was normal. Ferritin was 62 ng/mL. The white blood cell count (9.4 × 10^3^ cells/cm^3^) and hemoglobin (15.2 g/dL) were normal, and there was no lymphocytopenia. Hemoglobin A1c was 5.5%. A chest X-ray demonstrated pulmonary vessel cephalization and cardiomegaly. A computed tomography of the chest was performed to assess for pulmonary embolism (PE): there was a moderate right-sided pleural effusion, interstitial edema, and abdominal ascites and no PE; no calcifications were seen in the coronary arteries. His electrocardiogram was notable for sinus tachycardia without ST segment changes. A transthoracic echocardiogram demonstrated a severely enlarged left ventricle with severely reduced ejection fraction (EF) (LVEF < 20%) and severe global hypokinesis. The right ventricle was normal in size with severely reduced systolic function. No significant valvular dysfunction was identified. A cardiac magnetic resonance (CMR) with gadolinium contrast was performed for further myocardial tissue characterization and to exclude infiltrative/inflammatory cardiomyopathy. The CMR demonstrated a severely dilated left ventricle (left ventricular end diastolic volume index 166 mL/m^2^), with globally reduced systolic function and a left ventricular EF of 11%. The right ventricle was normal in size with severely reduced systolic function (right ventricular EF of 22%). LV wall thickness was normal (Figure 1), and there was no delayed enhancement with gadolinium contrast (Figure 2) to suggest scarring or infarction; a small patent foramen ovale was visualized. There is no evidence of myocardial iron overload. Finally, the coronary arteries had normal anatomical locations. These findings were consistent with idiopathic DCM.

## 5. Management

The patient responded well to intravenous diuresis and lost almost 10 kilograms over the course of 4 days. On hospital day 2, the patient was started on a low dose of valsartan 24 mg/sacubitril 26 mg. The patient was noted to develop borderline low blood pressure with systolic blood pressure near 90 mmHg; thus, further titration was held, and a beta-blocker was not started. He was safely discharged with close cardiology follow-up for medication titration.

## 6. Discussion

The Fine-Lubinsky syndrome, also called brachycephaly, deafness, cataract, microstomia, and mental retardation syndrome (BDCMMRS) [4], was first reported in 1983 by Dr. Fine and Dr. Lubinsky [5] as a disorder associated with craniofacial abnormalities, structural brain abnormalities including corpus callosum agenesis and hearing loss, developmental delay, ocular changes (glaucoma and cataracts), and other anatomical defects. Since that time, case reports have described 9 patients with this defect [1, 2, 4, 5–8]. The disease is thought to have an autosomal recessive inheritance pattern [9]. The mortality rate is unknown for this rare disorder; however, case reports have described death at 15 months of age in a patient with a febrile illness [8]. Few case reports have described cardiac anomalies in these patients: an infant who subsequently died at 15 months of age was found to have moderate pulmonary hypertension, a patent foramen ovale, hypertrophy, and a dilated right ventricle [8]. Thus, cardiac abnormalities and dysfunction may represent a previously unrecognized complication in this illness.

Heart failure in young adults is most commonly attributed to idiopathic dilated cardiomyopathy (DCM) [3]: in one study [2], over half of adults aged 20-39 years were diagnosed with idiopathic dilated (DCM). In the absence of hypertension, diabetes, and substance abuse/steroid use, viral myocarditis was considered as a possible etiology for the patient presentation. However, cardiac MRI findings did not show classic findings of acute myocarditis such as hyperemia, edema, or necrosis, thus making a viral myocarditis diagnosis less likely. Thus, the underlying cardiac dysfunction in this patient likely represents a previously unrecognized complication of the Fine-Lubinsky syndrome. However, it is also possible that this is idiopathic DCM unrelated to FLS. Genetic analyses are planned in the future for this patient to help resolve this question.

Finally, it is important to appreciate the morbidity of early-onset heart failure in the young adult population. In the CHARM programme study, the morbidity of heart failure diagnosis in patients aged 20-39 years was high: the 3-year mortality rate was 12%; these patients were more likely to have poor quality of life scores than in older age groups and required hospitalization for heart failure at a similar rate that to patients in the 60-69 years of age group. Thus, diagnosis of heart failure at a young age can have a significant impact on quality of life. Given this evidence, it is important to diagnose and institute guideline-based therapy in young patients as early as possible.

## 7. Follow-Up

This patient has not followed up yet.

## 8. Conclusions

We demonstrate here the first case report of heart failure in a young patient with a Fine-Lubinsky syndrome. The Fine-Lubinsky syndrome has only been associated with cardiac defects early in birth and has not been described in a young adult with this condition. Given the severity of heart failure observed in this case and the morbidity associated with a heart failure diagnosis at a young age, we recommend that providers consider screening patients with a Fine-Lubinsky syndrome for heart failure early in life.

## 9. Learning Objectives

- Describe the etiology of heart failure in young adults
- Demonstrate that Fine-Lubinsky may have previously unknown cardiac manifestations
- Evaluate whether there should be cardiac screening earlier in life in patients with a Fine-Lubinsky syndrome
